# Health-related quality of life outcomes of bioabsorbable Phasix Mesh versus permanent synthetic mesh following open ventral hernia repair: a systematic literature review and narrative synthesis

**DOI:** 10.3389/jaws.2026.16382

**Published:** 2026-05-11

**Authors:** Mia Weiss, Hakan Gürcan, Elise Aronitz, Haytham Gareer, John P. Fischer

**Affiliations:** 1 Becton, Dickinson and Company, Franklin Lakes, NJ, United States; 2 EVERSANA, Burlington, ON, Canada; 3 Division of Plastic Surgery, University of Pennsylvania Health System, Philadelphia, PA, United States

**Keywords:** permanent synthetic mesh, Phasix Mesh, poly-4-hydroxybutyrate, quality of life, ventral hernia repair

## Abstract

**Background:**

A hernia occurs when an organ or tissue protrudes through a weak spot in the surrounding muscle or connective tissue. The presence of a hernia, its surgical management, and the associated postoperative complications can substantially influence patient health-related quality of life (HRQoL). Ventral hernias often require mesh repair to reduce recurrence. While permanent synthetic meshes have historically been the standard of care, they have been associated with complications such as infection and chronic pain. As a result, patients are increasingly requesting alternatives, leading to growing interest in bioabsorbable meshes such as Phasix™ Mesh. This systematic literature review uses narrative synthesis to summarize reported HRQoL outcomes following open ventral hernia repair with Phasix™ Mesh or permanent synthetic mesh.

**Methods:**

Searches were conducted across multiple databases (i.e., MEDLINE®, Embase, Cochrane Central, and Cochrane Reviews) from 2013 to August 2024 using a peer-reviewed strategy. The primary outcome of the review was HRQoL, reported using the hernia-related quality of life survey (HerQLes).

**Results:**

Seven records of six independent studies met the inclusion criteria; four on Phasix™ Mesh and three on permanent synthetic mesh. Most studies were retrospective, with follow-up durations ranging from 12 to 66 months. A narrative synthesis was performed to summarize study design, patient characteristics, and HerQLes outcomes. Both mesh types showed improvements in HerQLes scores postoperatively. At the longest follow-up, the mean HerQLes postoperative scores were 75.25 for Phasix™ Mesh studies and 75.56 for permanent synthetic mesh studies. Quality of studies were moderate to high based on the Newcastle-Ottawa scale, supporting the reliability of findings.

**Conclusion:**

This study found that postoperative HerQLes scores improved with both Phasix™ Mesh and permanent synthetic meshes following open, ventral hernia repair. Single-arm studies of Phasix™ Mesh reported improvements in HerQLes scores up to 60 months post-surgery, including complex hernia cases. Early improvements in HerQLes scores were reported in permanent synthetic mesh studies, with reduced scores observed at the longest follow-up time points. Future large-scale, rigorously designed comparative studies are needed to confirm these findings, given no direct comparative studies are available.

## Introduction

A hernia is a medical condition where an organ, tissue, or structure in the body protrudes through a weak spot or opening in the surrounding muscle or connective tissue that usually holds it in place [1]. In 2019, more than 45 million (32.53 million prevalent and 13.02 million incident) cases of various abdominal hernias, such as inguinal, femoral, and abdominal, were recorded globally [2]. Ventral hernias account for an estimated 611,000 repairs annually in the United States of America (USA) [3], resulting in a cost of more than $3.2 billion annually to the USA [4]. This number is projected to grow due to rising risk factors such as an aging population, increasing obesity rates, and a higher incidence of abdominal surgeries unrelated to hernias [5].

Recurrence rates following hernia repair vary significantly depending on the type of hernia, with recurrence of ventral hernia repair occurring in 7.9%–25% of cases [6, 7]. These recurrences, along with complications, such as infections and other surgical site issues, can negatively affect patient outcomes and contribute to rising healthcare costs [8].

The presence of a hernia, its surgical management, and the associated postoperative complications can substantially influence patient health-related quality of life (HRQoL) [9]. The presence of a hernia may adversely affect various aspects of daily living, including sexual function, physical activity, and dietary habits, thereby impacting psychological wellbeing and social interactions [10]. Surgical intervention, particularly hernia repair, has been shown to improve patient outcomes [11]. Hernia repair is a technically demanding procedure that necessitates precise surgical execution to achieve optimal outcomes and enhance patient HRQoL [12]. Common surgical techniques include open, laparoscopic, and robotic approaches [13, 14].

The use of mesh has become the gold standard in surgical hernia repair due to its lower recurrence and reoperation rates compared to suture repair [15]. The first dedicated surgical mesh for hernia repair was introduced in 1955, which was composed of permanent synthetic non-degradable materials such as polyester, polypropylene, and expanded polytetrafluoroethylene [16]. Permanent synthetic meshes are valued for their mechanical strength and cost-effectiveness compared to biologic alternatives [17]. However, their use is not without the risk of complications, which may include chronic inflammation, low elasticity, infection, fistula formation, and persistent pain [14, 16]. Bioabsorbable mesh, is an alternative option constructed from non-permanent polymers such as poly-4-hydroxybutyrate (P4HB), which are gradually absorbed by the body [18]. In hernia repair, bioabsorbable meshes have demonstrated promising results compared to alternative meshes [18–21]. Bioabsorbable meshes offer the potential to mitigate long-term mesh related complications [18, 22].

Phasix™ Mesh (BD, Warwick, Rhode Island, USA) is a notable example of a bioabsorbable mesh, featuring an open monofilament scaffold composed entirely of P4HB [23]. This mesh is designed to degrade over 12–18 months into natural metabolites found in human tissues and recognized by the body, which can be eliminated through hydrolysis [24]. Many studies have supported the clinical efficacy and safety of Phasix™ Mesh, highlighting its ability to promote healthy tissue ingrowth and provoke a favorable host immune response [23, 25–28]. Preclinical and *in vitro* investigations have shown that Phasix™ Mesh facilitates rapid incorporation into host tissue and stimulates an early anti-inflammatory macrophage response, which is critical for effective tissue remodeling [29]. Phasix™ Mesh also provides long-term mechanical strength, more than twice that of other bioabsorbable meshes [24].

Recently, patient-reported outcomes (PROs) have become increasingly important in evaluating the effectiveness of hernia repair procedures [12]. While traditional clinical outcomes such as recurrence rates and surgical complications remain essential, they fail to fully capture the patient’s perspective [30]. Incorporating HRQoL tools that are tailored specifically for hernia can provide valuable insights into aspects such as physical, emotional, and social wellbeing, allowing for a more comprehensive evaluation of treatment success [31]. Although two recent systematic literature reviews (SLRs) have examined the clinical outcomes for Phasix™ Mesh [28, 32], there remains a notable paucity of studies summarizing the evidence on HRQoL. In this study, an SLR was conducted to identify studies reporting HRQoL outcomes for patients that underwent open, ventral hernia repair with permanent synthetic mesh or Phasix™ Mesh. HRQoL outcomes generated using the hernia-related quality of life survey (HerQLes) were described herein via narrative synthesis.

## Materials and methods

This review was conducted following the guidelines outlined in the Cochrane Handbook for Systematic Reviews of Interventions and adhered to the standards set by the Preferred Reporting Items for Systematic Reviews and Meta-Analyses (PRISMA) statement [33, 34]. The SLR protocol was not registered.

### Databases searched

All searches were conducted by a health librarian/medical information specialist. Using the Ovid® search interface, the following electronic databases were searched for studies reporting clinical and HRQoL outcomes: MEDLINE® and Epub Ahead of Print, In-Process, In-Data-Review & Other Non-Indexed Citations, Daily and Versions, Embase (1974–2024), EBM Reviews - Cochrane Central Register of Controlled Trials (1991-present), and EBM Reviews - Cochrane Database of Systematic Reviews (2005-present). Searches were performed on 1 August 2024. Search results were limited to records that were published after 2013 to align with the publication of the first Phasix™ Mesh studies [23, 24]. The full search strategy is provided in Supplementary Material 1.

### Inclusion and exclusion criteria

Studies were included if they reported clinical or HRQoL outcomes for patients undergoing open ventral hernia repair using either Phasix™ Mesh or permanent synthetic mesh. The predefined PICOS criteria are outlined in Table 1. If the study methods did not specify whether open, laparoscopic, or robotic-assisted surgical technique was used, it was assumed to be open surgery and included in the SLR. Studies that did not fulfill the inclusion criteria were excluded, with the reasons for exclusion documented. Additional filters were applied (i.e., only full text articles, published 2018 or later, minimum 12-month follow-up, and light/medium weight mesh) post-screening to increase homogeneity across included articles. Articles that passed this stage were further confirmed for inclusion during data extraction, which involved evaluating the study design, baseline characteristics of the study population, and reported efficacy outcomes.

**TABLE 1:** PICOS framework.

Items	Inclusion criteria	Exclusion criteria
Population	Human adults (≥18 years) undergoing open^a^ ventral hernia repair (primary ventral hernias: umbilical, epigastric, lumbar, spigelian, ventral; secondary ventral hernias: incisional, parastomal)	Patients under the age of 18 years old, those with inguinal hernia, and those undergoing laparoscopic or robotic-assisted surgeries
Intervention	Phasix™ Mesh (also known as P4HB mesh or poly-4-hydroxybutyrate mesh)	Any other (non-Phasix™ Mesh) bioabsorbable or biosynthetic meshes
Comparator	Synthetic (permanent) mesh	Biologic mesh
Outcome(s)	Any clinical^b^ or HRQoL measure including, but not limited to: hernia-related quality of life survey (HerQLes); European registry of abdominal wall hernias quality of life (EuraHS QoL); Carolinas comfort scale (CCS); abdominal hernia questionnaire (AHQ)^c^	Any non-HRQoL outcome
Study design	Clinical trials (randomized, non-randomized), single-arm studies, retrospective or prospective clinical studies, cohort studies, conference abstracts (past 2 years)	*In-vitro* studies, animal studies, and opinion/narrative pieces, case series, case reports, SLRs, meta-analyses, narrative reviews, editorials
Location	NA	NA
Language	English^d^	Non-English
Date limit	2013 onwards	Prior to 2013^e^

^a^ If the study methods did not specify whether open, laparoscopic, or robotic-assisted surgical technique was used, it was assumed to be open surgery and included in the SLR.

^b^ PICOS criteria included outcomes for both clinical and HRQoL outcomes; however, only HRQoL outcomes were of interest for this study.

^c^ Search captured all clinical outcomes to minimize the risk of loosing evidence; however, only those reporting the HRQoL outcomes were included in this study.

^d^ Search captured all languages, but non-English citations were excluded during screening.

^e^ Aligned with the publication of the first Phasix™ Mesh studies [23, 24].

Abbreviations: AHQ, abdominal hernia questionnaire; CCS, Carolinas comfort scale; EuraHS QoL, European registry of abdominal wall hernias quality of life; HerQLes, hernia-related quality of life survey; HRQoL, health-related quality of life; NA, not applicable; P4HB, poly-4-hydroxybutyrate; PICOS, population, intervention, comparator, outcome, and study design; SLR, systematic literature review.

### Study selection

Study selection was conducted using the systematic review platform DistillerSR (DistillerSR Inc., 2021, Ottawa, Canada). Two reviewers independently evaluated the study records, including titles and abstracts, to determine their eligibility. Reviewers recorded the reasons for excluding studies, and any disagreements between the two reviewers were resolved either through consensus or by consulting a third independent reviewer.

### Data extraction

Data extraction was conducted by a primary reviewer and independently confirmed by a second reviewer to ensure accuracy. Any disagreements between the two were resolved through discussion, or if necessary, by consulting a third independent reviewer. Data from the selected studies were gathered using standardized extraction templates created in Microsoft® Excel. The extracted study characteristics and outcomes included study information (i.e., author name, publication year, registration number, country of origin, follow-up duration, and study design), baseline population characteristics, treatment and disease details, and efficacy.

### Study quality

Risk of bias for the included studies was assessed using the Newcastle-Ottawa Scale (NOS) as it is specifically designed for observational studies (which comprised the majority of included studies) [35]. The assessment was completed in duplicate by two independent reviewers and any differences in judgments were resolved through discussion, or, if needed, by involving a third reviewer. With the NOS, the total score ranges from 0 to 9 stars, with studies scoring 7 or more considered high quality [35].

### Data synthesis

Although the SLR included studies reporting both clinical and HRQoL outcomes, HRQoL outcomes were the focus of the study described herein. A narrative synthesis was conducted which included detailed descriptions of study characteristics, methodologies, types of mesh used, and key findings. The interpretation of results considered the study populations, treatment factors, and methodological rigor. Additionally, a narrative overview of each study’s strengths and limitations was provided, along with responses to individual items from the risk of bias assessment to offer insight into the overall quality of the evidence. Quality of life results generated using the Hernia-Related Quality of Life Survey (HerQLes) were prioritized since this tool is validated for use in patients with hernia and results were consistently reported with sufficient detail across studies, thereby minimizing heterogeneity and facilitating robust comparisons.

## Results

### Literature search and screening

**FIGURE 1:**
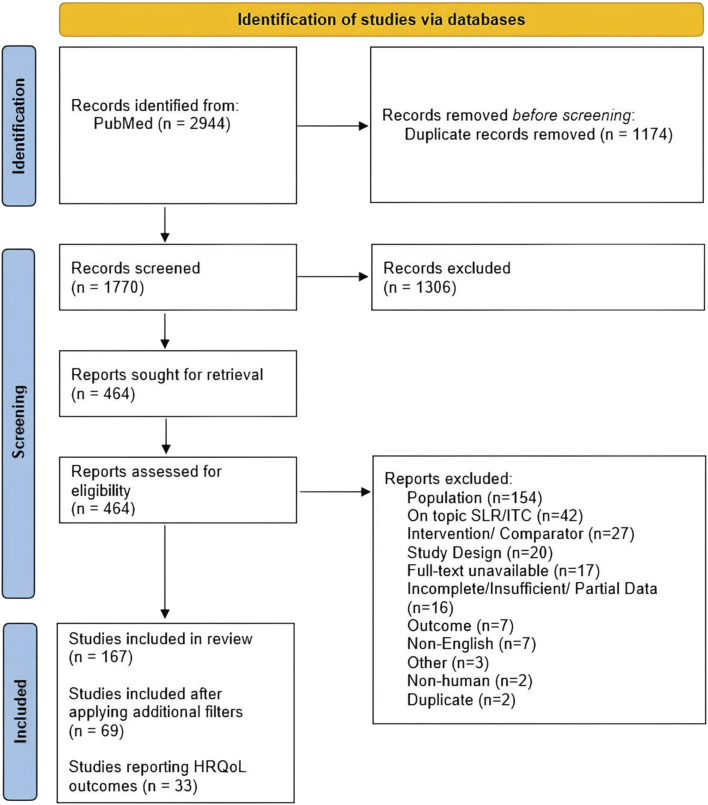
PRISMA flow diagram. Abbreviations: HRQoL, health-related quality of life; ITC, indirect treatment comparison; PRISMA, Preferred Reporting Items for Systematic Reviews and Meta-Analyses; SLR, systematic literature review.

The database searches yielded 2,944 records; after removing duplicates, 1,770 records were screened at the title and abstract phase, of which 1,306 were excluded. Of the remaining 464 records considered at the full-text screening phase, 297 were excluded for reasons as detailed in the PRISMA flow diagram (Figure 1) [34]. After completion of article screening and applying post-hoc filters to increase homogeneity across the included studies, an additional 98 articles were excluded, ultimately resulting in the inclusion of 69 articles. Of the 69 articles included, 33 reported HRQoL outcomes (with the remainder reporting clinical outcomes).

### HRQoL instruments

Of the 33 studies reporting HRQoL outcomes, 28 studies reported such outcomes using 12 different HRQoL scales; namely, abdominal hernia questionnaire (AHQ) [36–41], the Carolinas comfort scale (CCS) [42–44], EuroQol 5-dimension (EQ-5D) [4, 45–47], EuroQol visual analogue scale (EQ VAS) [4], HerQLes [4, 36, 39, 46, 48–50], numeric rating scale (NRS) [42], patient-reported outcomes measurement information system (PROMIS) [50], short-form 12-item survey (SF-12) [43, 51], short-form 36-item survey (SF-36) [52, 53], the visual analogue scale (VAS) [38, 43, 46, 54–60], the ventral hernia screening (VHS) [61], and verbal rating scale (VRS; Table 2) [62]. The remaining five articles reported HRQoL outcomes as the proportion of patients returning to usual activities or with chronic pain and/or swelling [63–67].

**TABLE 2:** HRQoL instruments used across included studies.

HRQoL instrument	Phasix™ Mesh (number of studies)		Permanent synthetic mesh (number of studies)	
	Pre-operative	Post-operative	Pre-operative	Post-operative
AHQ	4	5	1	1
CCS	1	1	1	2
EQ-5D	0	0	2	2
EQ VAS	0	0	2	2
HerQLes	4	4	3	3
NRS	0	0	1	1
PROMIS	0	0	1	1
SF-12	2	2	0	0
SF-36	0	0	2	2
VAS	3	4	3	6
VHS	0	1	0	0
VRS	0	0	0	1

Abbreviations: AHQ, abdominal hernia questionnaire; CCS, Carolinas comfort scale; EQ-5D, EuroQol 5-dimension; EQ VAS, EuroQol visual analogue scale; HerQLes, hernia-related quality of life survey; HRQoL = health-related quality of life; NRS, numeric rating scale; SF-12, short-form 12-item; SF-36, short-form 36-item; VAS, visual analogue scale; VHS, ventral hernia screening; VRS, verbal rating scale.

Among the studies that reported both pre-operative and post-operative HRQoL scores, all Phasix™ Mesh studies demonstrated improvements in HRQoL, regardless of the assessment instrument used [36, 37, 39–41, 43, 48, 49, 51, 54, 59]. Similarly, studies involving synthetic meshes consistently reported HRQoL improvements, independent of the measurement tool applied [4, 38, 42, 46, 50, 52, 53, 58].

The most commonly used HRQoL instruments across both Phasix™ Mesh and permanent synthetic mesh studies were the AHQ, HerQLes, and VAS (Table 2). Results derived using the AHQ were reported more often in Phasix™ Mesh studies relative to permanent synthetic mesh studies, whereas HerQLes and VAS were reported more evenly across studies of both mesh types (Table 2). The VAS is a validated tool used to measure pain intensity and is not specific to patients with hernia [68], whereas HerQLes is a validated, hernia-specific HRQoL instrument developed specifically for this patient population [69]. Its widespread adoption in hernia research supports its relevance and reliability [30, 70–72]. Therefore, only studies reporting HerQLes results were described further in this narrative review.

### Characteristics of studies reporting HerQLes outcomes

In total, seven records reporting HerQLes outcomes were included in this review, comprising six distinct studies and one secondary analysis based on one of the included studies. Four of the included studies evaluated Phasix™ Mesh and three assessed permanent synthetic mesh. The characteristics of the included studies are provided in Table 3. All studies were conducted in the United States and primarily employed retrospective designs, with one randomized controlled trial (RCT) [4] and one secondary analysis of a RCT [46].

**TABLE 3:** Study and patient characteristics of included studies.

Author, year	Registration number	Study design	Country	Follow up duration (months)	Patient population (n)	Proportion male, n (%)	BMI (kg/m^2^), mean (SD or range)	Mean age, years (SD or range)	Diabetic status, n (%)
Phasix™ Mesh									
Messa [48]	Protocol # 832515	Retrospective review	USA	Mean (range): 24 (12.2–41)	70	37 (52.8)	33.0 (20.3–53.3)	58 (23–81)	16 (23)
Christopher [36]	NR	Retrospective cohort study	USA	Mean (SD): 24.2 (12.9)	60	23 (38.3)	31 (26–44)	52.5 (45–66)	11 (18.3)
Christopher [49]	NR	Retrospective cohort study	USA	Median (IQR): 43.1 (38.2–49.1)	71	37 (52.1)	31 (25.8–38.8)	61.2 (50.5–68.5)	17 (23.9)
Talwar [39]	NR	Retrospective analysis of prospective assessments	USA	Median (IQR): 62.3 (57.8–66.6)	51	24 (47.1)	31.7 (24.1–38.7)	62.3 (50.8–67.6)	9 (17.6)
Permanent synthetic mesh									
Miller [46]	IDE# G120130, NCT#02451176	Post hoc analysis of RTC	USA	Total follow-up time: 24	51	29 (57)	31.7 (27–35)	63.4 (59–71)	8 (16)
Rosen [4]	NCT02451176	Multicenter single-blinded parallel RCT	USA	Total follow-up time: 24	126	61 (48)	32.3 (28–37)	63.7 (55–69)	29 (23)
Zolin [50]	NR	Retrospective analysis	USA	Median (IQR): 24 (12–48)	1,203	558 (46)	32 (28–36)	60 (52–67)	275 (23)

Abbreviations: BMI, body mass index; IDE, investigational device exemption; IQR, interquartile range; kg, kilogram; m, meter; n, number of patients; NCT, national clinical trial; NR, not reported; RCT, randomized controlled trial; SD, standard deviation; USA, United States of America.

Across studies, follow-up durations ranged from approximately 12–66 months, with reported follow-up durations spanning 12.2–66.6 months in Phasix™ Mesh studies and 12–48 months in permanent synthetic mesh studies. This variability in follow-up time points introduced significant heterogeneity and limited the ability to pool outcomes across studies. Sample sizes varied from 51 to 1,203 patients in permanent synthetic mesh studies, and from 51 to 71 patients in Phasix™ Mesh studies.

The mean patient age across studies ranged from the early 50s to mid-60s, and body mass index (BMI) values ranged from approximately 31–33 kg/m^2^. The proportion of male patients ranged from 38% to 57%, and the prevalence of diabetes ranged from 16% to 23.9%.

Hernia disease characteristics of included studies are presented in Table 4. The included studies primarily addressed ventral hernias, with some studies reporting detailed hernia location including umbilical, paramedian, or parastomal hernias. Hernia defect sizes were consistently large, with reported mean or median areas ranging from approximately 273 cm^2^–345 cm^2^. Varied reporting of hernia dimensions was noted, with lengths typically between 18–23 cm and widths between 13 and 16 cm. The patients included in these studies were complex, as all were diagnosed with large hernias (defined as having a defect of ≥10 cm in width or length, or an area of ≥78.5 cm^2^); such patients commonly experience high rates of recurrence and complications [73, 74].

**TABLE 4:** Hernia disease characteristics.

Author, year	Hernia type	Hernia size
Messa [48]	Ventral	Mean (range): 323 cm^2^ (25–972 cm^2^)
Christopher [36]	Incisional/ventral, umbilical, paramedian	Median (IQR): 300 (150–480) cm^2^
Christopher [49]	Ventral	Defect size area: 300 cm^2^ (180–444) Median (IQR) length: 20 (15–23) cm Median (IQR) width: 16 (12–23) cm
Talwar [39]	Ventral	Median (IQR): Area: 289 (150–440) cm^2^ Length: 18 (15–22) cm Width: 16 (11–23) cm
Miller [46]	Parastomal hernia, parastomal and midline	Median (IQR): Length: 21.0 (18.0–25.0) Width: 13.0 (10.5–15.0) Calculated area: 273 cm^2^
Rosen [4]	Ventral	Median (IQR): Length: 23 (16–25) cm Width: 14.0 (11–15) cm Calculated area: 322 cm^2^
Zolin [50]	Incisional, primary epigastric, primary umbilical, spigelian, lumbar	Median (IQR): Length: 23 (19–27) cm Width: 15 (2–19) cm Calculated area: 345 cm^2^

Abbreviations: cm, centimeter; IQR, interquartile range; n = number; NR, not reported; SD, standard deviation.

### HerQLes outcomes

HerQLes is a 12-item, 6-point Likert-style survey (with higher scores indicating better results) assessing HRQoL related to abdominal wall function in patients with hernia disease [75]. An increase of at least 15.6 points represents the minimal clinically important difference (MCID) when using the HerQLes [72].

**FIGURE 2:**
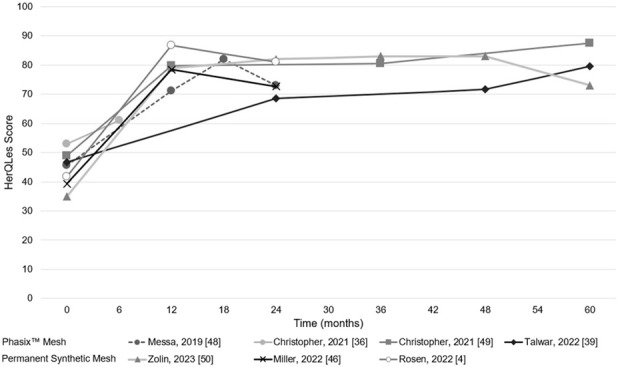
Visual depiction of HerQLes scores across included studies. Note: The following assumptions were made in order to present the above data visually: If a range of months was denoted in the study from which the HerQLes values were derived, the highest month was selected for the purpose of plotting the data. Christopher [36] did not report the time frame associated with the post-operative assessment; therefore, it was assumed that “post-operative” implied within the first 6 months of the procedure. Christopher [49] reported 36+ months for the post-operative time point for HerQLes assessment; this was plotted assuming the data point corresponded with 60-month follow-up. Messa [48] reported data for 18–24+ months which was plotted under 24 months. Abbreviations: HerQLes = hernia-related quality of life survey. Sources: Messa [48], Christopher [36], Christopher [49], Talwar [39], Zolin [50], Miller [46], Rosen [4].

A summary of HerQLes results spanning the included studies is presented in Table 5 and Figure 2. Both Phasix™ Mesh and permanent synthetic mesh studies demonstrated notable improvements in patient-reported HerQLes scores following hernia repair. However, the magnitude and trajectory of improvement varied between mesh types.

**TABLE 5:** Summary of HerQLes results across included studies.

Study	Pre-operative (baseline)		Post-operative			Mean difference
	N	Mean pre-operative score (SD)	N	Time point (months)	Mean post-operative score (SD)	
Phasix™ Mesh						
Messa [48]	59	45.6 (NR)	59	6–12	71.2 (NR)	**25.6**
			59	12–18	82.0 (NR)	**36.4**
			59	18–24+	73.0 (NR)	**27.4**
Christopher [36]	24	53.0 (NR)	24	NR	61.0 (NR)	8.0
Christopher [49]	56	48.9 (NR)	51	0–12	79.8 (NR)	**30.9**
			44	12–36	80.5 (NR)	**31.6**
			37	36+	87.5 (NR)	**38.6**
Talwar [39]	38	46.7 (26.6)	43	0–24	68.5 (25.2)	**21.8**
			24	36–48	71.6 (NR)	**23.9**
			43	60	79.5 (NR)	**30.8**
Permanent synthetic mesh						
Zolin [50]	768	35 (29.6)	768	12	79 (29.9)	**44**
			498	24	82 (29.9)	**47**
			349	36	83 (31.1)	**48**
			216	48	83 (31.1)	**48**
			153	60	73 (37.0)	**38**
Miller [46]	51	39.3 (47.6)	41	12	78.4 (44.1)	**39.1**
			45	24	72.7 (45.5)	**33.4**
Rosen [4]	126	41.79 (39.9)	108	12	86.81 (37.2)	**45.01**
			110	24	80.97 (37.5)	**39.17**

Bolding indicates mean difference in score surpasses the minimal clinically important difference of 15.6 points.

Abbreviations: HerQLes, hernia-related quality of life survey; n, number of patients; NR, not reported; SD, standard deviation.

In studies evaluating Phasix™ Mesh, improvements in HerQLes scores were observed across multiple follow-up time points relative to baseline. The average increase in HerQLes scores across all time points compared to baseline was approximately 27.5 points. Improvements were evident as early as 0–12 months post-operatively (i.e., mean increase of 25.6 points compared to baseline in Messa 2019 [48] and mean increase of 30.9 points compared to baseline in Christopher, 2021 [49]) and continued to increase through longer-term follow-up, reaching a maximum mean difference of 38.6 points compared to baseline at 36+ months [49]. The longest follow-up timepoint reported was 60 months, whereby a 30.8 points increase in HerQLes score was observed relative to baseline [39].

Permanent synthetic mesh studies demonstrated numerically large and immediate improvements, with an average HerQLes score increase of 42.4 points compared to baseline scores across all time points. While cohorts treated with permanent synthetic mesh experienced early HRQoL improvements (i.e., within the first 12 months), HerQLes scores declined over time in all three studies [4, 46, 50]. For example, in the Zolin 2023 study [50], by the 60 months, the mean difference in HerQLes scores decreased from 48 to 38. Similarly, Rosen 2022 [4] and Miller 2022 [46] reported substantial early improvements (45.01 and 39.1 points increase in HerQLes compared to baseline at 12 months, respectively), which declined to respectively 39.17 and 33.4 points improvements at 24 months.

Overall, considering the longest follow-up in each study, the mean post-operative HerQLes score was 75.25 in Phasix™ Mesh studies and 75.56 in permanent synthetic mesh studies.

### Risk of bias assessment

The NOS was used to assess the risk of bias among included studies. A summary of these assessments is provided in Supplementary Material 2. The methodological quality of included studies varied between mesh types.

Studies evaluating Phasix™ Mesh generally demonstrated moderate methodological quality, with total quality scores ranging from 5 to 7 out of 9 and a corresponding low-to-medium risk of bias. Among these, Messa 2019 achieved a score of 7 and low risk of bias, with strengths in exposure ascertainment, outcome assessment, and follow-up procedures, although it lacked a well-selected non-exposed cohort and did not adjust for secondary risk factors [48]. The two Christopher studies and the Talwar study shared similar limitations, including the absence of adjustments for key confounders and weaknesses in cohort selection, resulting in medium risk of bias and slightly lower scores [36, 39, 49]. On the other hand, the permanent synthetic mesh studies were generally of high quality, with all three studies receiving low risk of bias ratings. Rosen achieved a score of 9, indicating robust methodology across all domains, including comprehensive adjustment for confounders [4]. Miller and Zolin also performed well, each scoring 7, though they shared some limitations in non-exposed cohort selection and adjustment for secondary risk factors [46, 50].

## Discussion

The objective of this study was to conduct a SLR for studies reporting HRQoL outcomes in patients undergoing ventral hernia repair with either Phasix™ Mesh or permanent synthetic meshes culminating in a narrative synthesis of results generated via the HerQLes. A total of seven records met the pre-specified inclusion criteria: four evaluating Phasix™ Mesh and three assessing permanent synthetic mesh. Both Phasix™ Mesh and permanent synthetic mesh studies showed improvements in HerQLes scores post-operatively relative to baseline. Phasix™ Mesh studies demonstrated improvements in HerQLes scores up to 60 months post-surgery (average increase of 27.5 points in HerQLes compared to baseline). The improvements in HRQoL with Phasix™ Mesh were maintained even in studies involving complex hernia cases [36]. Permanent synthetic mesh studies showed early improvements (average 42.4 points increase in HerQLes compared to baseline) which tended to decline over time (the mean difference in HerQLes scores decreased by 10 points in Zolin [50], 5.84 points in Miller [46], and 5.7 points in Rosen [4] over their duration of follow-up). This declining trend raises questions about the sustainability of early gains in the permanent synthetic mesh studies. Nonetheless, these findings suggest that both permanent synthetic mesh and Phasix™ Mesh impart clinically significant improvements in HerQLes scores relative to baseline. Improvements in HerQLes scores were reported through the longest follow-up assessments in studies evaluating Phasix™ Mesh. In studies evaluating permanent synthetic mesh, HerQLes scores at the longest follow-up assessments were lower than those reported at earlier post-operative time points. These observations are descriptive and should be interpreted cautiously given the lack of comparative data.

The gains in HRQoL described herein are particularly important since, as a bioabsorbable mesh, Phasix™ Mesh minimizes the footprint within the abdominal wall [60, 76], which may result in a reduced risk of chronic pain and foreign body sensation compared to the use of permanent synthetic mesh [77]. Furthermore, the results of two independent meta-analyses have reported on the safety and efficacy of Phasix™ Mesh, underscoring it as a promising alternative to permanent synthetic mesh, especially for patients at higher risk of foreign body reactions, by providing a natural and bioabsorbable material [28, 32]. In a time where the selection of surgical mesh for ventral hernia repair is increasingly guided by patient-centered outcomes, offering patients a variety of mesh options that improve HRQoL over the long-term is of critical importance and facilitates shared decision-making which, in turn, leads to patient satisfaction [78].

Several methodological strengths helped enhance the robustness of the findings. A comprehensive systematic literature review was conducted to identify the relevant evidence for this study. To ensure the relevance and reliability of the patient-reported outcomes presented, data extraction prioritized values derived from a validated, hernia-specific quality of life instrument (i.e., HerQLes). However, this approach may limit the comprehensiveness of HRQoL assessment and introduce outcome reporting bias. The inclusion of both baseline and post-operative data allowed for a comprehensive assessment of changes in HRQoL over time. Also, the alignment of follow-up durations across studies evaluating different mesh types supported the comparison of outcomes, minimizing the risk of bias associated with time-dependent variables. Finally, risk of bias assessments indicated that the included studies were overall of good methodological quality, further supporting the credibility of the findings.

This review is subject to some limitations that should be considered when interpreting the findings. The absence of prospective protocol registration may limit reproducibility and is acknowledged as a limitation; however, efforts were made to mitigate this risk by adhering to established methodological guidance, including the PRISMA statement and relevant principles from the Cochrane Handbook for Systematic Reviews of Interventions [33, 34]. The small number of included studies, combined with heterogeneity in the characteristics of their recruited patients, limits the generalizability of the results. Additionally, incomplete data reporting in some studies hindered comprehensive data extraction and quantitative data synthesis. The reliance on narrative synthesis, necessitated by the high heterogeneity and incomplete data reporting among included studies, restricts the ability to perform meta-analyses and/or direct between-study comparisons. Nonetheless, this narrative review of data provides an initial assessment that warrants further investigation via head-to-head comparative studies. To reduce bias, the review was conducted using a predefined and transparent methodology, including systematic literature searches, explicit inclusion and exclusion criteria, and reliance exclusively on published data. Data extraction and synthesis were descriptive and non-comparative in nature, and no selective interpretation of outcomes was performed.

To enhance the reliability and interpretability of HRQoL outcomes, future research should prioritize the inclusion of more homogenous patient populations and standardized HRQoL assessment tools. This approach would reduce heterogeneity across studies, allowing for more meaningful comparisons and quantitative synthesis of findings. By minimizing heterogeneity in both participant characteristics and measurement tools, researchers can better isolate the effects of interventions on HRQoL and draw reliable conclusions that are more applicable to clinical practice.

In summary, this study found that post-operative HRQoL, as assessed using HerQLes, improved for patients with ventral hernia treated with either Phasix™ Mesh or permanent synthetic mesh. These findings reflect within study observations derived from heterogeneous, noncomparative data. Studies evaluating bioabsorbable Phasix™ Mesh showed improvements in HerQLes scores across multiple follow-up time points extending up to 60 months post-surgery. As a bioabsorbable material, Phasix™ Mesh is designed to resorb over time and therefore does not result in a permanent abdominal mesh footprint. Early improvements in HerQLes scores were reported in permanent synthetic mesh studies, with reduced scores observed at the longest follow-up time points. The absence of head-to-head comparisons precludes any conclusions regarding comparative effectiveness. Large-scale, rigorously designed comparative studies are needed to establish definitive comparative effectiveness of Phasix™ Mesh relative to permanent synthetic mesh.

## Glossary

AHQ: abdominal hernia questionnaire
BMI: body mass index
CCS: Carolinas comfort scale
EQ VAS: EuroQol visual analogue scale
EQ-5D: EuroQol 5-dimension
EuraHS QoL: European registry of abdominal wall hernias quality of life
HerQLes: hernia-related quality of life survey
HRQoL: health-related quality of life
IDE: investigational device exemption
IQR: interquartile range
ITC: indirect treatment comparison
MCID: minimal clinically important difference
NA: not applicable
NCT: national clinical trial
NOS: Newcastle-Ottawa scale
NR: not reported
NRS: numeric rating scale
PICOS: population, intervention, comparison, outcome, study design
PRISMA: preferred reporting items for systematic reviews and meta-analyses
PRO: patient-reported outcome
PROMIS: patient-reported outcomes measurement information system
RCT: randomized controlled trial
SD: standard deviation
SF-12: short-form 12-item survey
SF-36: short-form 36-item survey
SLR: systematic literature review
USA: United States of America
VAS: visual analogue scale
VHS: ventral hernia screening
VRS: verbal rating scale
