# Supplementary material for: Health-related quality of life outcomes of bioabsorbable Phasix Mesh versus permanent synthetic mesh following open ventral hernia repair: a systematic literature review and narrative synthesis
Source: J Abdom Wall Surg. 2026 May 11;5:16382. doi: 10.3389/jaws.2026.16382 (PMC13199133; doi:10.3389/jaws.2026.16382)
Supplement: Supplementary file 1 [file Table1.docx]

**Supplementary Material 1: Search Strategy**

| **#** | **Searches** | **Results** |
| --- | --- | --- |
| 1 | ((Incisional Hernia/ or exp Hernia, Ventral/ or Herniorrhaphy/ or (Surgical Stomas/ and Hernia/) or exp Hernia, Abdominal/) and (ventral$ or incisional$ or epigastric or epi-gastric or postoperative$ or post-operative$ or post-op or postop or exomphalos or omphalocele? or cicatricial$ or scar? or umbilic$ or semilunar or semi-lunar or Spiegel$ or Spieghel$ or Spigel$ or stoma$ or parastoma$ or para-stoma$ or lumba$ or lumbo$ or gastroschis#s or gastro-schis#s or (congenit$ adj3 fissur$ adj3 (abdominal adj1 cavit$3)) or inguinal or groin$ or iliac or femoral$ or obturator$ or inguinofemoral$ or inguino-femoral$ or sciatic$ or umbilic$ or exomphalos or cicatricial$ or scar$ or ((abdominal$ or abdomen$) adj4 herni$) or (open adj2 (surg$ or repair$ or herniorrhaph$ or herniorraph$ or hernio-rrhaph$ or hernio-rraph$ or hernioplast$ or hernio-plast$))).ti,ab.) or (herni$ adj4 (ventral$ or incisional$ or epigastric or epi-gastric or postoperative$ or post-operative$ or post-op or postop or exomphalos or omphalocele? or cicatricial$ or scar? or umbilic$ or semilunar or semi-lunar or Spiegel$ or Spieghel$ or Spigel$ or stoma$ or parastoma$ or para-stoma$ or lumba$ or lumbo$ or gastroschis#s or gastro-schis#s or (congenit$ adj3 fissur$ adj3 (abdominal adj1 cavit$3)) or inguinal or groin$ or iliac or femoral$ or obturator$ or inguinofemoral$ or inguino-femoral$ or sciatic$ or umbilic$ or exomphalos or cicatricial$ or scar$ or ((abdominal$ or abdomen$) adj4 herni$) or (open adj2 (surg$ or repair$ or herniorrhaph$ or herniorraph$ or hernio-rrhaph$ or hernio-rraph$ or hernioplast$ or hernio-plast$)))).ti,ab,kw,kf. [VENTRAL/INCISIONAL HERNIA TERMS] | 121926 |
| 2 | (Surgical Mesh/ and Phasix$.ti,ab,kw,kf.) or (((mesh$2 or "open monofilament scaffold") adj3 (biosynthetic$ or bioabsorbab$ or bioresorbab$ or bio-synthetic$ or bio-absorbab$ or bio-resorbab$ or ((fully or completely or totally or long-term or longterm or "100 per cent" or "100 percent") adj1 (resorbab$ or re-sorbab$ or absorbab$ or resorb$3 or absorb$3)) or P4HB or poly-4-hydroxybutyrate or "4-hydroxybutyrate")) or Phasix$).ti,ab,kw,kf. [PHASIX MESH TERMS] | 989 |
| 3 | (exp Surgical Mesh/ and (synthetic$ or permanent$ or metal$ or composite? or compositepolymer? or composite-polymer? or polypropylene or poly-propylene).ti,ab,kw,kf.) or ((((mesh$2 or "open monofilament scaffold" or AWL? or OMS? or sling?) adj3 (synthetic$ or permanent$ or metal$ or composite? or compositepolymer? or composite-polymer? or polypropylene or poly-propylene)) and (surgical$ or biologic$ or hernia?)) or "multi-purpose-surgical-meshcollagenbioabsorbable" or "multi-purpose-surgical-meshsynthetic-polymerbioabsorbable" or "multipurpose-surgical-meshcollagenbioabsorbable" or "multipurpose-surgical-meshsynthetic-polymerbioabsorbable" or "bio-a-tissue-reinforcement-prosthes#s").ti,ab,kw,kf. [SURGICAL SYNTHETIC MESH TERMS] | 16604 |
| 4 | (3D-Max-Mesh$2 or 3DMax$ or 4DDOME$ or Adhesix$ or AIGISRx$ or AlloDerm$ or AlloMax$ or Angimesh$2 or BioA-mesh$2 or (Biodesign adj1 "surgical mesh$2") or (C-Qur adj1 "surgical mesh$2") or C-QUR-CentriFX-Mesh$2 or C-QUR-Mosaic-Mesh$2 or CICAT$ or CollaMend$ or Composix$ or Crurasoft$ or DermaMatrix$ or DermMatrix$ or Dual-Mesh$2 or DualMesh$2 or Dulex$ or Dynamesh$2 or EpiDisc$ or Evolution-P3EM$ or FasLata$ or Flex-HD$ or FlexHD$ or FortaGen$ or Fortisan$ or Fortisan-Fabric$ or Glucamesh$2 or GORE-Infinit-mesh$2 or Gore-Tex-Dual-Mesh$2 or Gore-Tex-DualMesh$2 or Hermesh$2 or Hertra$ or HI-TEX$ or Infinit-mesh$2 or IntePro-Lite$ or InteXen$ or (Kugel adj2 mesh$2) or Kugel-Patch$2 or Mersilene-Polyester-Fiber-Suture$ or Mersuture-mesh$2 or MotifMesh$2 or MycroMesh$2 or NEOVEIL$ or Omyra-Mesh$2 or Optilene-Mesh-LP$ or Orthomesh$2 or Ovitex-PRS$ or Parietene$ or Parietex$ or Pelvicol$ or Pelvisoft$ or Pelvitex$ or PerFix$ or Peri-Strips-Dry$ or Permacol$ or ((PHS$2 or Proceed$2 or Polyform$ or Prolene$ or Surgimesh$2 or TIGR$2 or Veritas$) adj1 "surgical mesh$2") or ((Polysoft$ or Prolene$ or Soft$2 or Surgipro$ or Veritas$ or ProteGen$) adj1 mesh$2) or ((Polypropylene or Prolene$) adj1 "hernia system$2") or Physiomesh$2 or (PolySoft$ adj1 "hernia patch$2") or ProGrip$ or Prolene-3D-Patch$ or Prolene-Soft$ or ProLite$ or Restorelle$ or Sepramesh$2 or SoftMesh$2 or Strattice$ or Surgimesh$2 or (Surgipro$ adj2 (mesh$2 or patch$ or plug$)) or Symbotex$ or TELAMax$ or TephaFLEX$ or TIGR-Matrix$ or TIGR-Mesh$2 or TiLoop-Bra$ or TiMESH-light$ or TissueMend$ or Trelex$ or Tutomesh$2 or Tutopatch$ or Ultrapro$ or Ventralex$ or Ventralight$ or Ventrio$ or Vitamesh$2 or Vypro$ or collamend-fm-implant$ or dynamatrix$ or enduragen$ or galaflex$ or galaform-3d$ or gore-bio-a-fistula-plug$2 or gore-bio-a-hernia-plug$2 or gore-enform$ or kerecis-bioabsorbable-mesh$2 or matristem$ or miromesh$2 or "multi-purpose-surgical-meshcollagenbioabsorbab$" or "multi-purpose-surgical-meshsynthetic-polymerbioabsorbab$" or orthowrap$ or "pelvic-organ-prolapse-stress-urinary-incontinence-surgical-mesh$2" or proformix$ or seamguard$ or surgisis$ or "synthetic-polymer-bioabsorbable-multi-purpose-surgical-mesh$2" or tenomend$ or trelliform$ or vivosorb$ or xenform$ or xenmatrix$ or x-repair$ or "biodesign-hernia-mesh$2" or dextile$ or dualmesh-biomaterial$2 or duatene$ or gentrix$ or parietex$ or Versatex$ or Gilbert-plug$ or (Ultrapro$ adj1 (mesh$2 or advance$ or plug$)) or ((Apogee$ or Elevate$ or Perigee$ or ProteGen$) adj1 (mesh$2 adj sling?)) or I-STOP-TOMS$ or InVance$ or Perigee-system$ or Seratom$).ti,ab,kw,kf. [MESH DEVICES TERMS] | 30490 |
| 5 | 2 or 3 or 4 | 45460 |
| 6 | 1 and 5 [PHASIX OR SYNTHETIC MESH & VENTRAL/INCISIONAL HERNIA & OPEN REPAIR ] | 9459 |
| 7 | (exp Randomized Controlled Trial/ or (randomized controlled trial or controlled clinical trial).pt. or (randomized or placebo or randomly or trial or groups).ti,ab. or drug therapy.fs.) not (exp Animals/ not humans.sh.) [RCTs – MEDLINE sensitive Filter – Cochrane HSSS, 2023 revision] | 7581521 |
| 8 | exp Randomized Controlled Trials as Topic/ or Clinical Trial, Phase II/ or Clinical Trial, Phase III/ or Clinical Trial, Phase IV/ or (equivalence trial or pragmatic clinical trial).pt. or (randomised or randomi#ation? or RCT or placebo$ or ((singl$ or doubl$ or trebl$ or tripl$) adj (mask$ or blind$ or dumm$)) or ((study or trial or CT) adj3 (phase 2 or phase 2a or phase 2b or phase 2c or phase II or phase IIa or phase IIb or phase IIc or phase 3 or phase 3a or phase 3b or phase 3c or phase III or phase IIIa or phase IIIb or phase IIIc or "phase? 2/3" or "phase? II/III" or phase 4 or phase 4a or phase 4b or phase 4c or phase IV or phase IVa or phase IVb or phase IVc or "phase? 3/4" or "phase? III/IV")) or open-label$ or openlabel$).tw,kw,kf. [PHASE 2-4, OPEN LABEL - ADDITIONAL TERMS TO SUPPLEMENT RCTs FILTER] | 2727505 |
| 9 | Cohort Studies/ or Comparative Study/ or Follow-Up Studies/ or Prospective Studies/ or Risk Factors/ or cohort.mp. or compared.mp. or groups.mp. or multivariate.mp. [NON-RANDOMIZED STUDIES – MEDLINE Filter - sensitive, Furlan,2006] | 22968203 |
| 10 | Comparative study/ or Follow-Up Studies/ or Time Factors/ or (preoperat$ or pre-operat$).mp. or (chang$ or evaluat$ or reviewed or prospective$ or retrospective$ or baseline or cohort or case series).tw. [OBSERVATIONAL STUDIES – MEDLINE Filter – max specificity, Fraser, 2006] | 28432486 |
| 11 | Non-Randomized Controlled Trials As Topic/ or Controlled Before-After Studies/ or Interrupted Time Series Analysis/ or Historically Controlled Study/ or exp Case-Control Studies/ or Cross-Sectional Studies/ or Observational Study/ or Control Groups/ or (((cohort or concurrent or non-concurrent or incidence or follow-up or followup or longitudinal or prospective or retrospective or nonrandom$ or non-random$ or quasi-random$ or quasi-experiment$ or quasirandom$ or quasiexperiment$ or pretest or posttest or pre-test or post-test or "before after" or CBA or (historical$ adj2 control$) or case-control$ or case-comparison or case-compeer or case-referrent or case-referent or case-base or casecontrol$ or casecomparison or casecompeer or casereferrent or casereferent or casebase or cross-section$ or crosssection$ or prevalence) adj3 (stud$ or design? or trial?)) or (control$ adj2 group$) or non-RCT or nonRCT or nRCT or real-world or "real life evidence" or RWE or regist$ or (ITS adj2 (stud$ or design$ or trial?)) or (interrupted adj2 time adj2 series)).tw,kw,kf. [ADDITIONAL TERMS TO SUPPLEMENT NRS FILTERS] | 9976555 |
| 12 | ((single-arm or singlearm) adj2 (trial? or stud$ or design?)).tw,kw,kf. [SINGLE-ARM STUDIES – MEDLINE] | 24908 |
| 13 | or/7-12 [ALL CLINICAL TRIALS] | 39599202 |
| 14 | (systematic review or systematic literature review or systematic scoping review or systematic narrative review or systematic qualitative review or systematic evidence review or systematic quantitative review or "systematic meta-review" or systematic critical review or systematic mixed studies review or systematic mapping review or systematic cochrane review or "systematic search and review" or systematic integrative review).ti. not comment.pt. not (protocol or protocols).ti. not MEDLINE.st. | 359108 |
| 15 | (1469-493X or 1361-6137).is. and review.pt. | 29583 |
| 16 | systematic review.pt. | 277789 |
| 17 | or/14-16 [Ovid Expert Searches: SLR filter 2019] | 644795 |
| 18 | (meta-analy$ or metanaly$ or metaanaly$ or met-analy$).mp,pt. or review.pt. [SLR & MA - modified; Montori, 2004 - Balanced Filter ] | 7197785 |
| 19 | Network Meta-Analysis/ or ((network adj1 (MA or MAs)) or (NMA or NMAs or MTC or MTCs or MAIC or MAICs or ITC or ITCs or STC or STCs) or indirect$ compar$ or (indirect treatment$ adj1 compar$) or (mixed treatment$ adj1 compar$) or (multiple treatment$ adj1 compar$) or (multi-treatment$ adj1 compar$) or simultaneous$ compar$ or mixed comparison?).tw,kw,kf. [Additional terms for MA, NMA, ITC] | 72765 |
| 20 | (cochrane or evidence report or systematic reviews).jw. | 67002 |
| 21 | (systematic overview$ or evidence-based review$ or evidence-based overview$ or (evidence adj3 (review$ or overview$ or synthes$)) or meta-review$ or meta-overview$ or meta-synthes$ or metareview$ or metaoverview$ or metasynthes$ or rapid review$ or "review of reviews" or umbrella review?).tw,kw,kf. [Additional terms for synonyms for systematic reviews] | 219510 |
| 22 | or/17-21 [SLR & MA FILTERS - Combined - MEDLINE] | 7427717 |
| 23 | Single-Case Studies as Topic/ or Case Reports/ or (((case series or case-base or case) adj3 (stud$ or design?)) or casebase? or (case adj1 (stud$ or report?))).tw,kw,kf. [Remove case and case-series studies - MEDLINE] | 4133546 |
| 24 | (13 or 22) not 23 [ALL CLINICAL TRIALS & SLRs & MAs - Case Studies removed] | 41572842 |
| 25 | 6 and 24 [ALL SURGICAL MESH & ABDOMINAL HERNIA & ALL CLINICAL TRIALS] | 7085 |
| 26 | Quality-Adjusted Life Years/ | 57132 |
| 27 | (quality adjusted or adjusted life year$).ti,ab,kf. | 70388 |
| 28 | (qaly$ or qald$ or qale$ or qtime$).ti,ab,kf. | 50671 |
| 29 | (illness state$1 or health state$1).ti,ab,kf. | 26330 |
| 30 | (hui or hui1 or hui2 or hui3).ti,ab,kf. | 5868 |
| 31 | (multiattribute$ or multi attribute$).ti,ab,kf. | 3288 |
| 32 | (utility adj3 (score$1 or valu$ or health$ or cost$ or measur$ or disease$ or mean or gain or gains or index$)).ti,ab,kf. | 61589 |
| 33 | utilities.ti,ab,kf. | 27807 |
| 34 | (eq-5d or eq5d or eq-5 or eq5 or euro qual or euroqual or euro qual5d or euroqual5d or euro qol or euroqol or euro qol5d or euroqol5d or euro quol or euroquol or euro quol5d or euroquol5d or eur qol or eurqol or eur qol5d or eur qol5d or eur?qul or eur?qul5d or euro$ quality of life or European qol).ti,ab,kf. | 68886 |
| 35 | (euro$ adj3 (5 d or 5d or 5 dimension$ or 5dimension$ or 5 domain$ or 5domain$)).ti,ab,kf. | 20809 |
| 36 | (sf36$ or sf 36$ or sf thirtysix or sf thirty six).ti,ab,kf. | 92127 |
| 37 | (time trade off$1 or time tradeoff$1 or tto or timetradeoff$1).ti,ab,kf. | 6628 |
| 38 | quality of life/ and ((quality of life or qol) adj (score$1 or measure$1)).ti,ab,kf. | 56239 |
| 39 | quality of life/ and ec.fs. | 80888 |
| 40 | quality of life/ and (health adj3 status).ti,ab,kf. | 36854 |
| 41 | (quality of life or qol).ti,ab,kf. and Cost-Benefit Analysis/ | 29837 |
| 42 | ((qol or hrqol or quality of life).ti,kf. or *quality of life/) and ((qol or hrqol$ or quality of life) adj2 (increas$ or decrease$ or improv$ or declin$ or reduc$ or high$ or low$ or effect or effects or worse or score or scores or change$1 or impact$1 or impacted or deteriorat$)).ab. | 153701 |
| 43 | Cost-Benefit Analysis/ and (cost-effectiveness ratio$ and (perspective$ or life expectanc$)).ti,ab,kf. | 8259 |
| 44 | *quality of life/ and (quality of life or qol).ti. | 185823 |
| 45 | quality of life/ and ((quality of life or qol) adj3 (improv$ or chang$)).ti,ab,kf. | 166599 |
| 46 | quality of life/ and health-related quality of life.ti,ab,kf. | 141190 |
| 47 | models,economic/ | 15440 |
| 48 | or/26-47 [Arber 2017 Utilities filter - max sensitivity - MEDLINE] | 748189 |
| 49 | exp Health Status Indicators/ or ("European Quality of Life questionnaire 5D" or "disability adjusted life" or "sickness impact profile" or daly$ or (short form 36 or shortform 36 or shortform thirtysix or shortform thirty six or short form thirtysix or short form thirty six or sfthirtysix or sfthirty-six) or (sf6 or sf 6 or short form 6 or shortform 6 or sf six or sfsix or shortform six or short form six) or (sf12 or sf 12 or short form 12 or shortform 12 or sf twelve or sftwelve or shortform twelve or short form twelve) or (sf6D or sf 6D or short form 6D or shortform 6D or sf six D or sfsixD or shortform six D or short form six D) or (sf20 or sf 20 or short form 20 or shortform 20 or sf twenty or sftwenty or shortform twenty or short form twenty) or (hql or hqol or h qol or hrqol or hr qol) or (hye or hyes) or health$ year$ equivalent$ or disutili$ or rosser or (quality adj2 (wellbeing or well-being)) or qwb or standard gamble$ or "Hernia-Related Quality of Life Survey" or HerQles or "European Registry of Abdominal Wall Hernias Quality of Life" or "EuraHS QoL" or "Carolinas Comfort Scale" or CCS or "Abdominal Hernia-Q" or AHQ).ti,ab. or (SG and gamble).ab. [ADDITIONAL TERMS TO SUPPLEMENT Arber 2017 Utilities filter] | 607753 |
| 50 | 48 or 49 [UTILITIES] | 1208315 |
| 51 | 6 and 50 [ALL SURGICAL MESH & ABDOMINAL HERNIA & UTILITIES] | 583 |
| 52 | 25 or 51 [ALL SURGICAL MESH & ABDOMINAL HERNIA & ALL CLINICAL TRIALS/UTILITIES] | 7111 |
| 53 | (exp Child/ or exp Infant/) not ((exp Adult/ or Adolescent/) and (exp Child/ or exp Infant/)) [CHILDREN <13 REMOVED - MEDLINE] | 3454117 |
| 54 | exp Animals/ not (exp Animals/ and Humans/) [ANIMAL STUDIES ONLY - REMOVE - MEDLINE] | 17056074 |
| 55 | (address or autobiography or bibliography or biography or comment or dictionary or directory or editorial or "expression of concern" or festschrift or historical article or interactive tutorial or lecture or legal case or legislation or news or newspaper article or patient education handout or personal narrative or portrait or video-audio media or webcast or (letter not (letter and randomized controlled trial))).pt. [Opinion publications - Remove -MEDLINE] | 5106587 |
| 56 | 52 not (53 or 54 or 55) [CHILDREN <13, ANIMAL STUDIES and OPINION PUBLICATIONS - REMOVED - MEDLINE] | 5387 |
| 57 | 56 use ppezv [MEDLINE results - ALL SURGICAL MESH] | 2134 |
| 58 | ((incisional hernia/ or umbilical hernia/ or spigelian hernia/ or abdominal wall hernia/ or herniorrhaphy/) and (ventral$ or incisional$ or epigastric or epi-gastric or postoperative$ or post-operative$ or post-op or postop or exomphalos or omphalocele? or cicatricial$ or scar? or umbilic$ or semilunar or semi-lunar or Spiegel$ or Spieghel$ or Spigel$ or stoma$ or parastoma$ or para-stoma$ or lumba$ or lumbo$ or gastroschis#s or gastro-schis#s or (congenit$ adj3 fissur$ adj3 (abdominal adj1 cavit$3)) or inguinal or groin$ or iliac or femoral$ or obturator$ or inguinofemoral$ or inguino-femoral$ or sciatic$ or umbilic$ or exomphalos or cicatricial$ or scar$ or ((abdominal$ or abdomen$) adj4 herni$) or (open adj2 (surg$ or repair$ or herniorrhaph$ or herniorraph$ or hernio-rrhaph$ or hernio-rraph$ or hernioplast$ or hernio-plast$))).ti,ab.) or (herni$ adj4 (ventral$ or incisional$ or epigastric or epi-gastric or postoperative$ or post-operative$ or post-op or postop or exomphalos or omphalocele? or cicatricial$ or scar? or umbilic$ or semilunar or semi-lunar or Spiegel$ or Spieghel$ or Spigel$ or stoma$ or parastoma$ or para-stoma$ or lumba$ or lumbo$ or gastroschis#s or gastro-schis#s or (congenit$ adj3 fissur$ adj3 (abdominal adj1 cavit$3)) or inguinal or groin$ or iliac or femoral$ or obturator$ or inguinofemoral$ or inguino-femoral$ or sciatic$ or umbilic$ or exomphalos or cicatricial$ or scar$ or ((abdominal$ or abdomen$) adj4 herni$) or (open adj2 (surg$ or repair$ or herniorrhaph$ or herniorraph$ or hernio-rrhaph$ or hernio-rraph$ or hernioplast$ or hernio-plast$)))).ti,ab,kw,kf. [VENTRAL/INCISIONAL HERNIA TERMS] | 118583 |
| 59 | (surgical mesh/ and Phasix$.ti,ab,kw,kf.) or (((mesh$2 or "open monofilament scaffold") adj3 (biosynthetic$ or bioabsorbab$ or bioresorbab$ or bio-synthetic$ or bio-absorbab$ or bio-resorbab$ or ((fully or completely or totally or long-term or longterm or "100 per cent" or "100 percent") adj1 (resorbab$ or re-sorbab$ or absorbab$ or resorb$3 or absorb$3)) or P4HB or poly-4-hydroxybutyrate or "4-hydroxybutyrate")) or Phasix$).ti,ab,kw,kf. [PHASIX MESH TERMS] | 989 |
| 60 | (exp surgical mesh/ and (synthetic$ or permanent$ or metal$ or composite? or compositepolymer? or composite-polymer? or polypropylene or poly-propylene).ti,ab,kw,kf.) or ((((mesh$2 or "open monofilament scaffold" or AWL? or OMS? or sling?) adj3 (synthetic$ or permanent$ or metal$ or composite? or compositepolymer? or composite-polymer? or polypropylene or poly-propylene)) and (surgical$ or biologic$ or hernia?)) or "multi-purpose-surgical-meshcollagenbioabsorbable" or "multi-purpose-surgical-meshsynthetic-polymerbioabsorbable" or "multipurpose-surgical-meshcollagenbioabsorbable" or "multipurpose-surgical-meshsynthetic-polymerbioabsorbable" or "bio-a-tissue-reinforcement-prosthes#s").ti,ab,kw,kf. [SURGICAL SYNTHETIC MESH TERMS] | 16604 |
| 61 | (3D-Max-Mesh$2 or 3DMax$ or 4DDOME$ or Adhesix$ or AIGISRx$ or AlloDerm$ or AlloMax$ or Angimesh$2 or BioA-mesh$2 or (Biodesign adj1 "surgical mesh$2") or (C-Qur adj1 "surgical mesh$2") or C-QUR-CentriFX-Mesh$2 or C-QUR-Mosaic-Mesh$2 or CICAT$ or CollaMend$ or Composix$ or Crurasoft$ or DermaMatrix$ or DermMatrix$ or Dual-Mesh$2 or DualMesh$2 or Dulex$ or Dynamesh$2 or EpiDisc$ or Evolution-P3EM$ or FasLata$ or Flex-HD$ or FlexHD$ or FortaGen$ or Fortisan$ or Fortisan-Fabric$ or Glucamesh$2 or GORE-Infinit-mesh$2 or Gore-Tex-Dual-Mesh$2 or Gore-Tex-DualMesh$2 or Hermesh$2 or Hertra$ or HI-TEX$ or Infinit-mesh$2 or IntePro-Lite$ or InteXen$ or (Kugel adj2 mesh$2) or Kugel-Patch$2 or Mersilene-Polyester-Fiber-Suture$ or Mersuture-mesh$2 or MotifMesh$2 or MycroMesh$2 or NEOVEIL$ or Omyra-Mesh$2 or Optilene-Mesh-LP$ or Orthomesh$2 or Ovitex-PRS$ or Parietene$ or Parietex$ or Pelvicol$ or Pelvisoft$ or Pelvitex$ or PerFix$ or Peri-Strips-Dry$ or Permacol$ or ((PHS$2 or Proceed$2 or Polyform$ or Prolene$ or Surgimesh$2 or TIGR$2 or Veritas$) adj1 "surgical mesh$2") or ((Polysoft$ or Prolene$ or Soft$2 or Surgipro$ or Veritas$ or ProteGen$) adj1 mesh$2) or ((Polypropylene or Prolene$) adj1 "hernia system$2") or Physiomesh$2 or (PolySoft$ adj1 "hernia patch$2") or ProGrip$ or Prolene-3D-Patch$ or Prolene-Soft$ or ProLite$ or Restorelle$ or Sepramesh$2 or SoftMesh$2 or Strattice$ or Surgimesh$2 or (Surgipro$ adj2 (mesh$2 or patch$ or plug$)) or Symbotex$ or TELAMax$ or TephaFLEX$ or TIGR-Matrix$ or TIGR-Mesh$2 or TiLoop-Bra$ or TiMESH-light$ or TissueMend$ or Trelex$ or Tutomesh$2 or Tutopatch$ or Ultrapro$ or Ventralex$ or Ventralight$ or Ventrio$ or Vitamesh$2 or Vypro$ or collamend-fm-implant$ or dynamatrix$ or enduragen$ or galaflex$ or galaform-3d$ or gore-bio-a-fistula-plug$2 or gore-bio-a-hernia-plug$2 or gore-enform$ or kerecis-bioabsorbable-mesh$2 or matristem$ or miromesh$2 or "multi-purpose-surgical-meshcollagenbioabsorbab$" or "multi-purpose-surgical-meshsynthetic-polymerbioabsorbab$" or orthowrap$ or "pelvic-organ-prolapse-stress-urinary-incontinence-surgical-mesh$2" or proformix$ or seamguard$ or surgisis$ or "synthetic-polymer-bioabsorbable-multi-purpose-surgical-mesh$2" or tenomend$ or trelliform$ or vivosorb$ or xenform$ or xenmatrix$ or x-repair$ or "biodesign-hernia-mesh$2" or dextile$ or dualmesh-biomaterial$2 or duatene$ or gentrix$ or parietex$ or Versatex$ or Gilbert-plug$ or (Ultrapro$ adj1 (mesh$2 or advance$ or plug$)) or ((Apogee$ or Elevate$ or Perigee$ or ProteGen$) adj1 (mesh$2 adj sling?)) or I-STOP-TOMS$ or InVance$ or Perigee-system$ or Seratom$).ti,ab,kw,kf. [MESH DEVICES TERMS] | 30490 |
| 62 | 59 or 60 or 61 | 45460 |
| 63 | 58 and 62 [PHASIX OR SYNTHETIC MESH & VENTRAL/INCISIONAL HERNIA & OPEN REPAIR ] | 9382 |
| 64 | exp randomized controlled trial/ or controlled clinical study/ or randomization/ or intermethod comparison/ or double blind procedure/ or human experiment/ or trial.ti. or ((evaluated or evaluate or evaluating or assessed or assess) and (compare or compared or comparing or comparison)).ab. or (random$ or placebo or compare or compared or comparison or (open adj label) or ((double or single or doubly or singly) adj (blind or blinded or blindly)) or parallel group$1 or (crossover or cross over) or ((assign$ or match or matched or allocation) adj5 (alternate or group$1 or intervention$1 or patient$1 or subject$1 or participant$1)) or (assigned or allocated) or (controlled adj7 (study or design or trial)) or (volunteer or volunteers)).ti,ab. | 19667313 |
| 65 | ((random$ adj sampl$ adj7 ("cross section$" or questionnaire$1 or survey$ or database$1)).ti,ab. not (comparative study/ or controlled study/ or randomi?ed controlled.ti,ab. or randomly assigned.ti,ab.)) or (cross-sectional study/ not (exp randomized controlled trial/ or controlled clinical study/ or controlled study/ or randomi?ed controlled.ti,ab. or control group$1.ti,ab.)) or ((((case adj control$) and random$) not randomi?ed controlled) or (nonrandom$ not random$) or "Random field$" or (random cluster adj3 sampl$)).ti,ab. or (systematic review not (trial or study)).ti. or ((review.ab. and review.pt.) not trial.ti.) or ("we searched".ab. and (review.ti. or review.pt.)) or ("update review" or (databases adj4 searched)).ab. or ((rat or rats or mouse or mice or swine or porcine or murine or sheep or lambs or pigs or piglets or rabbit or rabbits or cat or cats or dog or dogs or cattle or bovine or monkey or monkeys or trout or marmoset$1).ti. and animal experiment/) or (Animal experiment/ not (human experiment/ or human/)) | 6633844 |
| 66 | 64 not 65 [RCTs – Embase sensitive Filter – Cochrane HSSS, 2023 revision] | 17805456 |
| 67 | phase 2 clinical trial/ or phase 3 clinical trial/ or phase 4 clinical trial/ or (equivalence trial or pragmatic clinical trial).pt. or (randomised or randomi#ation? or RCT or placebo* or ((singl$ or doubl$ or trebl$ or tripl$) adj (mask$ or blind$ or dumm$)) or ((study or trial or CT) adj3 (phase 2 or phase 2a or phase 2b or phase 2c or phase II or phase IIa or phase IIb or phase IIc or phase 3 or phase 3a or phase 3b or phase 3c or phase III or phase IIIa or phase IIIb or phase IIIc or "phase? 2/3" or "phase? II/III" or phase 4 or phase 4a or phase 4b or phase 4c or phase IV or phase IVa or phase IVb or phase IVc or "phase? 3/4" or "phase? III/IV")) or open-label$ or openlabel$).tw,kw,kf. [PHASE 2-4, OPEN LABEL - ADDITIONAL TERMS TO SUPPLEMENT RCTs FILTER] | 2411534 |
| 68 | Clinical article/ or controlled study/ or major clinical study/ or prospective study/ or cohort.mp. or compared.mp. or groups.mp. or multivariate.mp. [NON-RANDOMIZED STUDIES– Embase Filter - sensitive, Furlan,2006] | 27903984 |
| 69 | Controlled study/ or Treatment outcome/ or Major clinical study/ or Clinical trial/ or (chang$ or evaluat$ or reviewed or baseline or (compare$ or compara$)).tw. [OBSERVATIONAL STUDIES – Embase Filter – max specificity, Fraser, 2006] | 36487450 |
| 70 | exp cohort analysis/ or exp case control study/ or controlled clinical trial/ or pretest posttest control group design/ or static group comparison/ or control group/ or retrospective study/ or longitudinal study/ or intervention study/ or family study/ or case study/ or time series analysis/ or cross-sectional study/ or comparative study/ or observational study/ or quasi experimental study/ or (((cohort or concurrent or non-concurrent or incidence or follow-up or followup or longitudinal or prospective or retrospective or nonrandom$ or non-random$ or quasi-random$ or quasi-experiment$ or quasirandom$ or quasiexperiment$ or pretest or posttest or pre-test or post-test or "before after" or CBA or (historical$ adj2 control$) or case-control$ or case-comparison or case-compeer or case-referrent or case-referent or case-base or casecontrol$ or casecomparison or casecompeer or casereferrent or casereferent or casebase or cross-section$ or crosssection$ or prevalence) adj3 (stud$ or design? or trial?)) or (control$ adj2 group$) or non-RCT or nonRCT or nRCT or real-world or "real life evidence" or RWE or regist$ or (ITS adj2 (stud$ or design$ or trial?)) or (interrupted adj2 time adj2 series)).tw,kw,kf. [ADDITIONAL TERMS TO SUPPLEMENT NRS FILTERS] | 17022746 |
| 71 | ((single-arm or singlearm) adj2 (trial? or stud$ or design?)).tw,kw,kf. [SINGLE-ARM STUDIES – Embase] | 24908 |
| 72 | or/66-71 [ALL CLINICAL TRIALS] | 45965840 |
| 73 | exp meta analysis/ or ((meta adj analy$) or metaanalys$).mp. or (systematic adj (review? or overview?)).tw. or (cancerlit or cochrane or embase or psychlit or psyclit or psychinfo or psycinfo or cinahl or cinhal or science citation index or bids or reference lists or bibliograph$ or hand-search$ or manual search$ or relevant journals).ab. | 1420605 |
| 74 | (data extraction or selection criteria).ab. and review.pt. | 78949 |
| 75 | or/73-74 [SLR & MA FILTER - Ovid Expert Searches: SLR filter 2019] | 1433279 |
| 76 | (meta-analy$ or metanaly$ or metaanaly$ or met-analy$).mp. or review.pt. [SLR & MA FILTER - modified and translated; Montori, 2004 - Balanced Filter ] | 7197785 |
| 77 | network meta-analysis/ or ((network adj1 (MA or MAs)) or (NMA or NMAs or MTC or MTCs or MAIC or MAICs or ITC or ITCs or STC or STCs) or indirect$ compar$ or (indirect treatment$ adj1 compar$) or (mixed treatment$ adj1 compar$) or (multiple treatment$ adj1 compar$) or (multi-treatment$ adj1 compar$) or simultaneous$ compar$ or mixed comparison?).tw,kw,kf. [Additional terms for MA, NMA, ITC] | 72765 |
| 78 | (cochrane or evidence report or systematic reviews).jw. | 67002 |
| 79 | (systematic overview$ or evidence-based review$ or evidence-based overview$ or (evidence adj3 (review$ or overview$ or synthes$)) or meta-review$ or meta-overview$ or meta-synthes$ or metareview$ or metaoverview$ or metasynthes$ or rapid review$ or "review of reviews" or umbrella review?).tw,kw,kf. [Additional terms for synonyms for systematic reviews] | 219510 |
| 80 | or/75-79 [SLR & MA FILTERS - Combined - Embase] | 7575006 |
| 81 | case study/ or family study/ or Case Report/ or (((case series or case-base or case) adj3 (stud$ or design?)) or casebase? or (case adj1 (stud$ or report?))).tw,kw,kf. [Remove case and case-series studies - Embase] | 6438003 |
| 82 | (72 or 80) not 81 [ALL CLINICAL TRIALS & SLRs & MAs - Case Studies removed] | 45366931 |
| 83 | 63 and 82 [ALL SURGICAL MESH & ABDOMINAL HERNIA & ALL CLINICAL TRIALS] | 6899 |
| 84 | Quality-Adjusted Life Year/ | 57132 |
| 85 | (quality adjusted or adjusted life year$).ti,ab,kw. | 70175 |
| 86 | (qaly$ or qald$ or qale$ or qtime$).ti,ab,kw. | 50578 |
| 87 | (illness state$1 or health state$1).ti,ab,kw. | 26184 |
| 88 | (hui or hui1 or hui2 or hui3).ti,ab,kw. | 5844 |
| 89 | (multiattribute$ or multi attribute$).ti,ab,kw. | 3278 |
| 90 | (utility adj3 (score$1 or valu$ or health$ or cost$ or measur$ or disease$ or mean or gain or gains or index$)).ti,ab,kw. | 60797 |
| 91 | utilities.ti,ab,kw. | 27637 |
| 92 | (eq-5d or eq5d or eq-5 or eq5 or euro qual or euroqual or euro qual5d or euroqual5d or euro qol or euroqol or euro qol5d or euroqol5d or euro quol or euroquol or euro quol5d or euroquol5d or eur qol or eurqol or eur qol5d or eur qol5d or eur?qul or eur?qul5d or euro$ quality of life or European qol).ti,ab,kw. | 69241 |
| 93 | (euro$ adj3 (5 d or 5d or 5 dimension$ or 5dimension$ or 5 domain$ or 5domain$)).ti,ab,kw. | 20736 |
| 94 | (sf36$ or sf 36$ or sf thirtysix or sf thirty six).ti,ab,kw. | 92359 |
| 95 | (time trade off$1 or time tradeoff$1 or tto or timetradeoff$1).ti,ab,kw. | 6603 |
| 96 | "quality of life"/ and ((quality of life or qol) adj (score$1 or measure$1)).ti,ab,kw. | 56184 |
| 97 | "quality of life"/ and ec.fs. | 80888 |
| 98 | "quality of life"/ and (health adj3 status).ti,ab,kw. | 36209 |
| 99 | (quality of life or qol).ti,ab,kw. and "cost benefit analysis"/ | 29949 |
| 100 | ((qol or hrqol or quality of life).ti,kw. or *"quality of life"/) and ((qol or hrqol$ or quality of life) adj2 (increas$ or decrease$ or improv$ or declin$ or reduc$ or high$ or low$ or effect or effects or worse or score or scores or change$1 or impact$1 or impacted or deteriorat$)).ab. | 164087 |
| 101 | Cost-Benefit Analysis/ and (cost-effectiveness ratio$ and (perspective$ or life expectanc$)).ti,ab,kw. | 8233 |
| 102 | *"quality of life"/ and (quality of life or qol).ti. | 185823 |
| 103 | "quality of life"/ and ((quality of life or qol) adj3 (improv$ or chang$)).ti,ab,kw. | 166622 |
| 104 | "quality of life"/ and health-related quality of life.ti,ab,kw. | 140817 |
| 105 | economic model/ | 15440 |
| 106 | or/84-105 [Arber 2017 Utilities filter - max sensitivity - EMBASE translation] | 756288 |
| 107 | exp health status indicator/ or exp "European Quality of Life 5 Dimensions questionnaire"/ or exp "Short Form 36"/ or ("European Quality of Life questionnaire 5D" or "disability adjusted life" or "sickness impact profile" or daly$ or (short form 36 or shortform 36 or shortform thirtysix or shortform thirty six or short form thirtysix or short form thirty six or sfthirtysix or sfthirty-six) or (sf6 or sf 6 or short form 6 or shortform 6 or sf six or sfsix or shortform six or short form six) or (sf12 or sf 12 or short form 12 or shortform 12 or sf twelve or sftwelve or shortform twelve or short form twelve) or (sf6D or sf 6D or short form 6D or shortform 6D or sf six D or sfsixD or shortform six D or short form six D) or (sf20 or sf 20 or short form 20 or shortform 20 or sf twenty or sftwenty or shortform twenty or short form twenty) or (hql or hqol or h qol or hrqol or hr qol) or (hye or hyes) or health$ year$ equivalent$ or disutili$ or rosser or (quality adj2 (wellbeing or well-being)) or qwb or standard gamble$ or "Hernia-Related Quality of Life Survey" or HerQles or "European Registry of Abdominal Wall Hernias Quality of Life" or "EuraHS QoL" or "Carolinas Comfort Scale" or CCS or "Abdominal Hernia-Q" or AHQ).ti,ab. or (SG and gamble).ab. [ADDITIONAL TERMS TO SUPPLEMENT Arber 2017 Utilities filter] | 649380 |
| 108 | 106 or 107 [UTILITIES] | 1226432 |
| 109 | 63 and 108 [ALL SURGICAL MESH & ABDOMINAL HERNIA & UTILITIES] | 594 |
| 110 | 83 or 109 [ALL SURGICAL MESH & ABDOMINAL HERNIA & ALL CLINICAL TRIALS/UTILITIES] | 6933 |
| 111 | (exp adolescent/ or exp child/ or exp infant/) not (exp adult/ and (exp adolescent/ or exp child/ or exp infant/)) [CHILDREN <18 REMOVE - Embase] | 4896569 |
| 112 | (exp animal/ or exp animal experimentation/ or exp animal model/ or exp animal experiment/ or nonhuman/ or exp vertebrate/) not (exp human/ or exp human experimentation/ or exp human experiment/) [ANIMAL STUDIES ONLY - REMOVE - EMBASE] | 12756143 |
| 113 | (editorial or note or short survey or tombstone).pt. or (letter.pt. not randomized controlled trial/) [OPINION PIECES REMOVE - Embase] | 5510134 |
| 114 | 110 not (111 or 112 or 113) [CHILDREN <18, ANIMAL STUDIES and OPINION PUBLICATIONS - REMOVED - Embase] | 6003 |
| 115 | conference abstract.pt. | 5203811 |
| 116 | 114 and 115 [CONFERENCE ABSTRACTS ONLY] | 910 |
| 117 | limit 116 to yr="2022 -Current" | 152 |
| 118 | 114 not 115 [CONFERENCE ABSTRACTS REMOVED] | 5093 |
| 119 | 117 or 118 [LAST 2 YRS OF ABSTRACTS RETAINED - ALL SURGICAL MESH] | 5245 |
| 120 | 119 use oemezd [Embase results - ALL SURGICAL MESH] | 2365 |
| 121 | ((Incisional Hernia/ or exp Hernia, Ventral/ or Herniorrhaphy/) and (ventral$ or incisional$ or epigastric or epi-gastric or postoperative$ or post-operative$ or post-op or postop or exomphalos or omphalocele? or cicatricial$ or scar? or umbilic$ or semilunar or semi-lunar or Spiegel$ or Spieghel$ or Spigel$ or stoma$ or parastoma$ or para-stoma$ or lumba$ or lumbo$ or gastroschis#s or gastro-schis#s or (congenit$ adj3 fissur$ adj3 (abdominal adj1 cavit$3)) or inguinal or groin$ or iliac or femoral$ or obturator$ or inguinofemoral$ or inguino-femoral$ or sciatic$ or umbilic$ or exomphalos or cicatricial$ or scar$ or ((abdominal$ or abdomen$) adj4 herni$) or (open adj2 (surg$ or repair$ or herniorrhaph$ or herniorraph$ or hernio-rrhaph$ or hernio-rraph$ or hernioplast$ or hernio-plast$))).ti,ab.) or (herni$ adj4 (ventral$ or incisional$ or epigastric or epi-gastric or postoperative$ or post-operative$ or post-op or postop or exomphalos or omphalocele? or cicatricial$ or scar? or umbilic$ or semilunar or semi-lunar or Spiegel$ or Spieghel$ or Spigel$ or stoma$ or parastoma$ or para-stoma$ or lumba$ or lumbo$ or gastroschis#s or gastro-schis#s or (congenit$ adj3 fissur$ adj3 (abdominal adj1 cavit$3)) or inguinal or groin$ or iliac or femoral$ or obturator$ or inguinofemoral$ or inguino-femoral$ or sciatic$ or umbilic$ or exomphalos or cicatricial$ or scar$ or ((abdominal$ or abdomen$) adj4 herni$) or (open adj2 (surg$ or repair$ or herniorrhaph$ or herniorraph$ or hernio-rrhaph$ or hernio-rraph$ or hernioplast$ or hernio-plast$)))).ti,ab,kw. [VENTRAL/INCISIONAL HERNIA TERMS] | 117987 |
| 122 | (Surgical Mesh/ and Phasix$.ti,ab,kw.) or (((mesh$2 or "open monofilament scaffold") adj3 (biosynthetic$ or bioabsorbab$ or bioresorbab$ or bio-synthetic$ or bio-absorbab$ or bio-resorbab$ or ((fully or completely or totally or long-term or longterm or "100 per cent" or "100 percent") adj1 (resorbab$ or re-sorbab$ or absorbab$ or resorb$3 or absorb$3)) or P4HB or poly-4-hydroxybutyrate or "4-hydroxybutyrate")) or Phasix$).ti,ab,kw. [PHASIX MESH TERMS] | 976 |
| 123 | (exp Surgical Mesh/ and (synthetic$ or permanent$ or metal$ or composite? or compositepolymer? or composite-polymer? or polypropylene or poly-propylene).ti,ab,kw.) or ((((mesh$2 or "open monofilament scaffold" or AWL? or OMS? or sling?) adj3 (synthetic$ or permanent$ or metal$ or composite? or compositepolymer? or composite-polymer? or polypropylene or poly-propylene)) and (surgical$ or biologic$ or hernia?)) or "multi-purpose-surgical-meshcollagenbioabsorbable" or "multi-purpose-surgical-meshsynthetic-polymerbioabsorbable" or "multipurpose-surgical-meshcollagenbioabsorbable" or "multipurpose-surgical-meshsynthetic-polymerbioabsorbable" or "bio-a-tissue-reinforcement-prosthes#s").ti,ab,kw. [SURGICAL SYNTHETIC MESH TERMS] | 16422 |
| 124 | (3D-Max-Mesh$2 or 3DMax$ or 4DDOME$ or Adhesix$ or AIGISRx$ or AlloDerm$ or AlloMax$ or Angimesh$2 or BioA-mesh$2 or (Biodesign adj1 "surgical mesh$2") or (C-Qur adj1 "surgical mesh$2") or C-QUR-CentriFX-Mesh$2 or C-QUR-Mosaic-Mesh$2 or CICAT$ or CollaMend$ or Composix$ or Crurasoft$ or DermaMatrix$ or DermMatrix$ or Dual-Mesh$2 or DualMesh$2 or Dulex$ or Dynamesh$2 or EpiDisc$ or Evolution-P3EM$ or FasLata$ or Flex-HD$ or FlexHD$ or FortaGen$ or Fortisan$ or Fortisan-Fabric$ or Glucamesh$2 or GORE-Infinit-mesh$2 or Gore-Tex-Dual-Mesh$2 or Gore-Tex-DualMesh$2 or Hermesh$2 or Hertra$ or HI-TEX$ or Infinit-mesh$2 or IntePro-Lite$ or InteXen$ or (Kugel adj2 mesh$2) or Kugel-Patch$2 or Mersilene-Polyester-Fiber-Suture$ or Mersuture-mesh$2 or MotifMesh$2 or MycroMesh$2 or NEOVEIL$ or Omyra-Mesh$2 or Optilene-Mesh-LP$ or Orthomesh$2 or Ovitex-PRS$ or Parietene$ or Parietex$ or Pelvicol$ or Pelvisoft$ or Pelvitex$ or PerFix$ or Peri-Strips-Dry$ or Permacol$ or ((PHS$2 or Proceed$2 or Polyform$ or Prolene$ or Surgimesh$2 or TIGR$2 or Veritas$) adj1 "surgical mesh$2") or ((Polysoft$ or Prolene$ or Soft$2 or Surgipro$ or Veritas$ or ProteGen$) adj1 mesh$2) or ((Polypropylene or Prolene$) adj1 "hernia system$2") or Physiomesh$2 or (PolySoft$ adj1 "hernia patch$2") or ProGrip$ or Prolene-3D-Patch$ or Prolene-Soft$ or ProLite$ or Restorelle$ or Sepramesh$2 or SoftMesh$2 or Strattice$ or Surgimesh$2 or (Surgipro$ adj2 (mesh$2 or patch$ or plug$)) or Symbotex$ or TELAMax$ or TephaFLEX$ or TIGR-Matrix$ or TIGR-Mesh$2 or TiLoop-Bra$ or TiMESH-light$ or TissueMend$ or Trelex$ or Tutomesh$2 or Tutopatch$ or Ultrapro$ or Ventralex$ or Ventralight$ or Ventrio$ or Vitamesh$2 or Vypro$ or collamend-fm-implant$ or dynamatrix$ or enduragen$ or galaflex$ or galaform-3d$ or gore-bio-a-fistula-plug$2 or gore-bio-a-hernia-plug$2 or gore-enform$ or kerecis-bioabsorbable-mesh$2 or matristem$ or miromesh$2 or "multi-purpose-surgical-meshcollagenbioabsorbab$" or "multi-purpose-surgical-meshsynthetic-polymerbioabsorbab$" or orthowrap$ or "pelvic-organ-prolapse-stress-urinary-incontinence-surgical-mesh$2" or proformix$ or seamguard$ or surgisis$ or "synthetic-polymer-bioabsorbable-multi-purpose-surgical-mesh$2" or tenomend$ or trelliform$ or vivosorb$ or xenform$ or xenmatrix$ or x-repair$ or "biodesign-hernia-mesh$2" or dextile$ or dualmesh-biomaterial$2 or duatene$ or gentrix$ or parietex$ or Versatex$ or Gilbert-plug$ or (Ultrapro$ adj1 (mesh$2 or advance$ or plug$)) or ((Apogee$ or Elevate$ or Perigee$ or ProteGen$) adj1 (mesh$2 adj sling?)) or I-STOP-TOMS$ or InVance$ or Perigee-system$ or Seratom$).ti,ab,kw. [MESH DEVICES TERMS] | 30296 |
| 125 | 122 or 123 or 124 | 45114 |
| 126 | 121 and 125 [PHASIX OR SYNTHETIC MESH & VENTRAL/INCISIONAL HERNIA & OPEN REPAIR ] | 9279 |
| 127 | (editorial or note or comment).pt. or (letter.pt. not randomized controlled trial/) [OPINION PIECES REMOVE - CENTRAL] | 5429058 |
| 128 | 126 not 127 [ALL SURGICAL MESH & ABDOMINAL HERNIA - OPINION PIECES REMOVED - CENTRAL] | 9128 |
| 129 | Conference proceeding.pt. [CONFERENCE ABSTRACTS/PROCEEDINGS] | 242948 |
| 130 | 128 and 129 [CONFERENCE ABSTRACTS ONLY] | 109 |
| 131 | limit 130 to yr="2022 -Current" | 10 |
| 132 | 128 not 129 [CONFERENCE ABSTRACTS REMOVED] | 9019 |
| 133 | 131 or 132 [ALL SURGICAL MESH & ABDOMINAL HERNIA - LAST 2 YRS OF ABSTRACTS RETAINED] | 9029 |
| 134 | 133 use cctr [ALL SURGICAL MESH - CENTRAL results] | 681 |
| 135 | (herni$ adj4 (ventral$ or incisional$ or epigastric or epi-gastric or postoperative$ or post-operative$ or post-op or postop or exomphalos or omphalocele? or cicatricial$ or scar? or umbilic$ or semilunar or semi-lunar or Spiegel$ or Spieghel$ or Spigel$ or stoma$ or parastoma$ or para-stoma$ or lumba$ or lumbo$ or gastroschis#s or gastro-schis#s or (congenit$ adj3 fissur$ adj3 (abdominal adj1 cavit$3)) or inguinal or groin$ or iliac or femoral$ or obturator$ or inguinofemoral$ or inguino-femoral$ or sciatic$ or umbilic$ or exomphalos or cicatricial$ or scar$ or ((abdominal$ or abdomen$) adj4 herni$) or (open adj2 (surg$ or repair$ or herniorrhaph$ or herniorraph$ or hernio-rrhaph$ or hernio-rraph$ or hernioplast$ or hernio-plast$)))).ti,ab,kw. [VENTRAL/INCISIONAL HERNIA TERMS] | 110181 |
| 136 | (((mesh$2 or "open monofilament scaffold") adj3 (biosynthetic$ or bioabsorbab$ or bioresorbab$ or bio-synthetic$ or bio-absorbab$ or bio-resorbab$ or ((fully or completely or totally or long-term or longterm or "100 per cent" or "100 percent") adj1 (resorbab$ or re-sorbab$ or absorbab$ or resorb$3 or absorb$3)) or P4HB or poly-4-hydroxybutyrate or "4-hydroxybutyrate")) or Phasix$).ti,ab,kw. [PHASIX MESH TERMS] | 976 |
| 137 | ((((mesh$2 or "open monofilament scaffold" or AWL? or OMS? or sling?) adj3 (synthetic$ or permanent$ or metal$ or composite? or compositepolymer? or composite-polymer? or polypropylene or poly-propylene)) and (surgical$ or biologic$ or hernia?)) or "multi-purpose-surgical-meshcollagenbioabsorbable" or "multi-purpose-surgical-meshsynthetic-polymerbioabsorbable" or "multipurpose-surgical-meshcollagenbioabsorbable" or "multipurpose-surgical-meshsynthetic-polymerbioabsorbable" or "bio-a-tissue-reinforcement-prosthes#s").ti,ab,kw. [SURGICAL SYNTHETIC MESH TERMS] | 11838 |
| 138 | (3D-Max-Mesh$2 or 3DMax$ or 4DDOME$ or Adhesix$ or AIGISRx$ or AlloDerm$ or AlloMax$ or Angimesh$2 or BioA-mesh$2 or (Biodesign adj1 "surgical mesh$2") or (C-Qur adj1 "surgical mesh$2") or C-QUR-CentriFX-Mesh$2 or C-QUR-Mosaic-Mesh$2 or CICAT$ or CollaMend$ or Composix$ or Crurasoft$ or DermaMatrix$ or DermMatrix$ or Dual-Mesh$2 or DualMesh$2 or Dulex$ or Dynamesh$2 or EpiDisc$ or Evolution-P3EM$ or FasLata$ or Flex-HD$ or FlexHD$ or FortaGen$ or Fortisan$ or Fortisan-Fabric$ or Glucamesh$2 or GORE-Infinit-mesh$2 or Gore-Tex-Dual-Mesh$2 or Gore-Tex-DualMesh$2 or Hermesh$2 or Hertra$ or HI-TEX$ or Infinit-mesh$2 or IntePro-Lite$ or InteXen$ or (Kugel adj2 mesh$2) or Kugel-Patch$2 or Mersilene-Polyester-Fiber-Suture$ or Mersuture-mesh$2 or MotifMesh$2 or MycroMesh$2 or NEOVEIL$ or Omyra-Mesh$2 or Optilene-Mesh-LP$ or Orthomesh$2 or Ovitex-PRS$ or Parietene$ or Parietex$ or Pelvicol$ or Pelvisoft$ or Pelvitex$ or PerFix$ or Peri-Strips-Dry$ or Permacol$ or ((PHS$2 or Proceed$2 or Polyform$ or Prolene$ or Surgimesh$2 or TIGR$2 or Veritas$) adj1 "surgical mesh$2") or ((Polysoft$ or Prolene$ or Soft$2 or Surgipro$ or Veritas$ or ProteGen$) adj1 mesh$2) or ((Polypropylene or Prolene$) adj1 "hernia system$2") or Physiomesh$2 or (PolySoft$ adj1 "hernia patch$2") or ProGrip$ or Prolene-3D-Patch$ or Prolene-Soft$ or ProLite$ or Restorelle$ or Sepramesh$2 or SoftMesh$2 or Strattice$ or Surgimesh$2 or (Surgipro$ adj2 (mesh$2 or patch$ or plug$)) or Symbotex$ or TELAMax$ or TephaFLEX$ or TIGR-Matrix$ or TIGR-Mesh$2 or TiLoop-Bra$ or TiMESH-light$ or TissueMend$ or Trelex$ or Tutomesh$2 or Tutopatch$ or Ultrapro$ or Ventralex$ or Ventralight$ or Ventrio$ or Vitamesh$2 or Vypro$ or collamend-fm-implant$ or dynamatrix$ or enduragen$ or galaflex$ or galaform-3d$ or gore-bio-a-fistula-plug$2 or gore-bio-a-hernia-plug$2 or gore-enform$ or kerecis-bioabsorbable-mesh$2 or matristem$ or miromesh$2 or "multi-purpose-surgical-meshcollagenbioabsorbab$" or "multi-purpose-surgical-meshsynthetic-polymerbioabsorbab$" or orthowrap$ or "pelvic-organ-prolapse-stress-urinary-incontinence-surgical-mesh$2" or proformix$ or seamguard$ or surgisis$ or "synthetic-polymer-bioabsorbable-multi-purpose-surgical-mesh$2" or tenomend$ or trelliform$ or vivosorb$ or xenform$ or xenmatrix$ or x-repair$ or "biodesign-hernia-mesh$2" or dextile$ or dualmesh-biomaterial$2 or duatene$ or gentrix$ or parietex$ or Versatex$ or Gilbert-plug$ or (Ultrapro$ adj1 (mesh$2 or advance$ or plug$)) or ((Apogee$ or Elevate$ or Perigee$ or ProteGen$) adj1 (mesh$2 adj sling?)) or I-STOP-TOMS$ or InVance$ or Perigee-system$ or Seratom$).ti,ab,kw. [MESH DEVICES TERMS] | 30296 |
| 139 | 136 or 137 or 138 | 41036 |
| 140 | 135 and 139 [PHASIX OR SYNTHETIC MESH & VENTRAL/INCISIONAL HERNIA & OPEN REPAIR ] | 8409 |
| 141 | 140 use coch [ALL SURGICAL MESH - CDSR results] | 2 |
| 142 | 57 or 120 or 134 or 141 [ALL SURGICAL MESH - ALL DATABASES results] | 5182 |
| 143 | limit 142 to yr="2013-Current" | 2944 |
