# Supplementary material for: Health-related quality of life outcomes of bioabsorbable Phasix Mesh versus permanent synthetic mesh following open ventral hernia repair: a systematic literature review and narrative synthesis
Source: J Abdom Wall Surg. 2026 May 11;5:16382. doi: 10.3389/jaws.2026.16382 (PMC13199133; doi:10.3389/jaws.2026.16382)
Supplement: Supplementary file 2 [file Table2.docx]

**Supplementary Material 2: Risk of bias assessment of included studies**

| **Author, year** | **Selection** | | | | **Comparability** | | **Outcome** | | | **Total quality score (out of 9)** | **Risk of bias** |
| --- | --- | --- | --- | --- | --- | --- | --- | --- | --- | --- | --- |
|  | Representativeness of the exposed cohort | Selection of the non-exposed cohort | Ascertainment of exposure | Demonstration that outcome of interest was not present at start of study | Adjust for the most important risk factors | Adjust for other risk factors | Assessment of Outcome | follow-up length | Loss to follow up rate |  |  |
| **Phasix™ Mesh** | | | | | | | | | | | |
| Messa, 2019 [1] | 1 | 0 | 1 | 1 | 1 | 0 | 1 | 1 | 1 | 7 | Low |
| Christopher, 2021a [2] | 1 | 0 | 1 | 1 | 0 | 0 | 1 | 1 | 0 | 5 | Medium |
| Christopher, 2021b [3] | 1 | 0 | 1 | 1 | 0 | 0 | 1 | 1 | 1 | 6 | Medium |
| Talwar, 2022 [4] | 1 | 0 | 1 | 1 | 0 | 0 | 1 | 1 | 1 | 6 | Medium |
| **Synthetic mesh** | | | | | | | | | | | |
| Miller, 2022 [5] | 1 | 0 | 1 | 1 | 1 | 0 | 1 | 1 | 1 | 7 | Low |
| Rosen, 2022 [6] | 1 | 1 | 1 | 1 | 1 | 1 | 1 | 1 | 1 | 9 | Low |
| Zolin, 2023 [7] | 1 | 0 | 1 | 1 | 1 | 0 | 1 | 1 | 1 | 7 | Low |

**References:**

1. Messa CAI, Kozak G, Broach RB, Fischer JP. When the Mesh Goes Away: An Analysis of Poly-4-Hydroxybutyrate Mesh for Complex Hernia Repair. Plastic and Reconstructive Surgery – Global Open. 2019;7(11):e2576.

2. Christopher AN, Morris MP, Jia H, Broach R, Fischer JP. Resorbable Synthetic Ventral Hernia Repair in Contaminated Fields: Outcomes with Poly-4-Hydroxybutyrate Mesh. Plastic and Reconstructive Surgery. 2021;148(6):1367-75.

3. Christopher AN, Morris MP, Patel V, Mellia JA, Fowler C, Messa CA, et al. An evaluation of clinical and quality of life outcomes after ventral hernia repair with poly-4-hydroxybutyrate mesh. Hernia. 2021;25(3):717-26.

4. Talwar AA, Perry NJ, McAuliffe PB, Desai AA, Thrippleton S, Broach RB, et al. Shifting the Goalpost in Ventral Hernia Care: 5-year Outcomes after Ventral Hernia Repair with Poly-4-hydroxybutyrate Mesh. Hernia. 2022;26(6):1635-43.

5. Miller BT, Krpata DM, Petro CC, Beffa LRA, Carbonell AM, Warren JA, et al. Biologic vs Synthetic Mesh for Parastomal Hernia Repair: Post Hoc Analysis of a Multicenter Randomized Controlled Trial. J Am Coll Surg. 2022;235(3):401-9.

6. Rosen MJ, Krpata DM, Petro CC, Carbonell A, Warren J, Poulose BK, et al. Biologic vs Synthetic Mesh for Single-stage Repair of Contaminated Ventral Hernias: A Randomized Clinical Trial. JAMA Surg. 2022;157(4):293-301.

7. Zolin SJ, Krpata DM, Petro CC, Prabhu AS, Rosenblatt S, Rosen S, et al. Long-term Clinical and Patient-Reported Outcomes After Transversus Abdominis Release With Permanent Synthetic Mesh: A Single Center Analysis of 1203 Patients. Ann Surg. 2023;277(4):e900-e6.
